# Supplementary material for: A New Regioselective Synthesis of the Cysteine-Rich Peptide Linaclotide
Source: Molecules. 2023 Jan 19;28(3):1007. doi: 10.3390/molecules28031007 (PMC9919235; doi:10.3390/molecules28031007)
Supplement: Supplementary file 1 [file molecules-28-01007-s001.zip › molecules-2156774-supplementary.pdf]

Supporting Information

A New Regioselective Synthesis of the Cysteine-Rich Peptide Linaclotide

Zhonghao Qiu [1,2], Xiandong Dai [1], Chongxu Fan[1], Ying Cao[1], Zirui Lv [1], Xingyong Liang[2,*] and Fanhua Meng [1,*]

[1] State Key Laboratory of NBC Protection for Civilian, Beijing 102205, China
[2] School of Chemistry Engineering, Sichuan University of Science & Engineering, Zigong 643000, China
* Correspondence: mfh027@163.com (F. M.), levesonk@163.com (X. L.)

Table of Contents

1. Synthesis of Fmoc- L -Cys(S-oNBn)-OH 1

1.1 Preparation of Fmoc-L-Cys(S-oNBn)-OH 1

1.2 ^{1}H NMR and MS Spectra of Fmoc-L-Cys(S-oNBn)-OH 2

2. Synthesis of the Peptide Drug Vasopressin 3

2.1 Preparation of Linear Vasopressin Precursor 3

2.2 Preparation of Vasopressin 4

1. Synthesis of Fmoc- L -Cys(S-oNBn)-OH

1.1 Preparation of Fmoc-L-Cys(S-oNBn)-OH

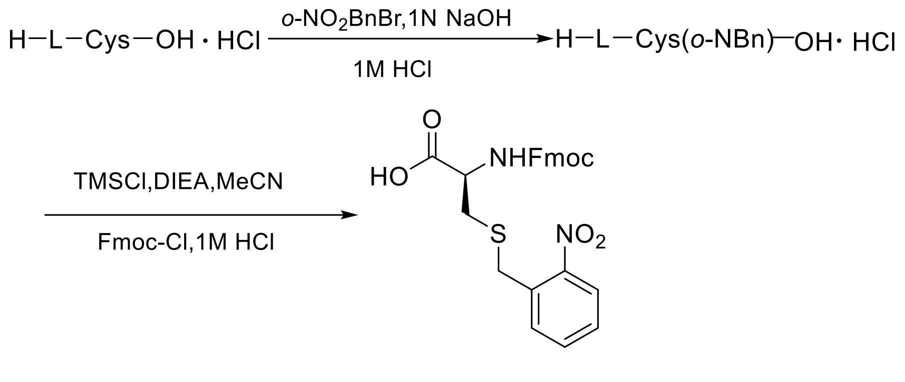

Figure S1 Synthesis of Fmoc- L -Cys(S-oNBn)-OH

To a 250 ml round bottom flask was sequentially added H-L-Cys-OH·HCl (7.9 g, 60 mmol), 100 ml 2N precooled NaOH aqueous solution. The mixture was stirred at 0 °C for 0.5 h, then 2-nitrobenzyl bromide (12.9 g, 75 mmol) was added and stirring was continued for another 5 h when the reaction was completed monitoring by thin-layer chromatography on GF254 silica gel plates (0.2 mm), the developing solvent was n-Butanol, acetic acid and H_2O (4 : 1 : 1,*v*/*v*/*v*), and the R_f value was about 0.4 . The mixture was filtered under reduced pressure and the filtrate was collected. The pH of filtrate was adjusted to 5 with acetic acid and incubation at room for hours afforded many white solids. The white solids were filtered and washed with 50% ethanol, dried by phosphorus pentoxide in vacuum desiccator for 3-4 days. H-L-Cys(o-NBn)-OH·HCl was obtained in 77% yield (10 g).

Crystalline H-L-Cys(o-NBn)-OH·HCl (2.5 g, 8.5 mmol) was suspended in dry MeCN (75 ml) in the dark at ambient temperature. Hünig's base (7.5 ml, 42.5 mmol) and TMSCl (1.35 ml, 10.625 mmol) were added, and the suspension was stirred for 0.5 h in an ice bath. After the reaction mixture cleared, Fmoc-Cl (2 g, 8.5 mmol) dissolved in 15 ml MeCN was added dropwise about 15 min. The reaction mixture was stirred for hours at 0 °C until the reaction was completed monitoring by thin-layer chromatography on GF254 silica gel plates (0.2 mm), the developing solvent was chloroform, methanol and acetic acid (9 : 1 : 0.2,*v*/*v*/*v*), and the R_f value was about 0.65 . Which diluted with EtOAc (250 ml), then washed with 1M HCl (250 ml ×3) and brine (250 ml). The organic layer was dried (Na_2SO_4), filtered and concentrated in vacuo. The resulting viscous oil was dissolved in Et_2O (50 mL) and precipitated with heptane (50 mL). A foamy amorphous solid was produced in 72% yield (3 g) by placing the precipitate on high vacuum: ^1H NMR (400 MHz, DMSO) δ 12.89 (s, 1H), 8.01 (d, J = 8.1 Hz, 1H), 7.89 (d, J = 7.5 Hz, 2H), 7.74 (t, J = 8.0 Hz, 2H), 7.67 (t, J = 7.4 Hz, 1H), 7.59 (d, J = 7.6 Hz, 1H), 7.53 (t, J = 7.6 Hz, 1H), 7.41 (t, J = 7.3 Hz, 2H), 7.32 (t, J = 7.4 Hz, 2H), 4.37 – 4.17 (m, 3H), 4.16 – 3.99 (m, 3H), 2.84 (dd, J = 17.4, 13.8 Hz, 1H), 2.76 – 2.63 (m, 1H). HRMS (ESI): *m*/*z* calculated for $[M+Na]^+$: 501.1096; found: 501.1091.

1.2 ^{1}H NMR and MS Spectra of Fmoc-L-Cys(S-oNBn)-OH

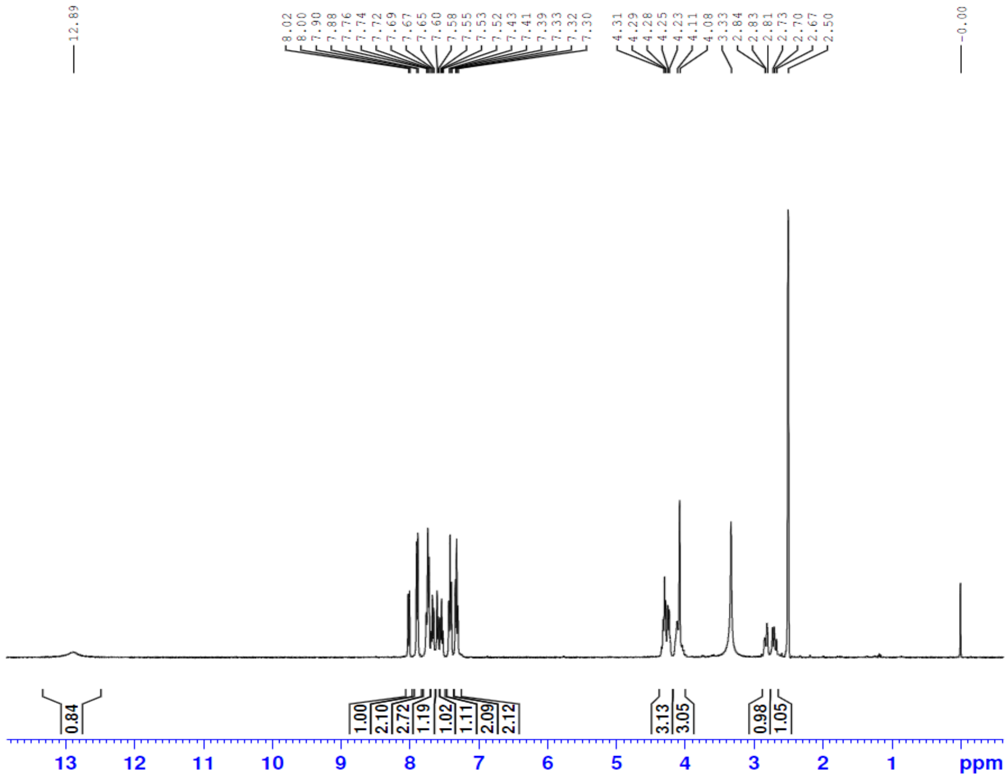

Figure S2 ^{1}H NMR Spectra of Fmoc-L-Cys(S-oNBn)-OH

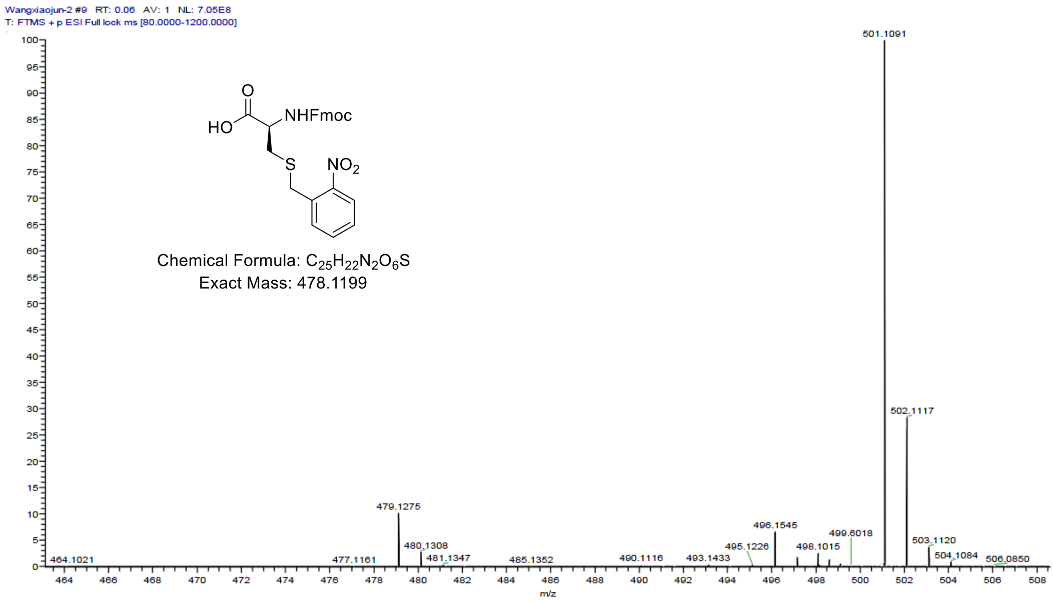

Figure S3 MS Spectra of Fmoc-L-Cys(S-oNBn)-OH

2.Synthesis of the Peptide Drug Vasopressin

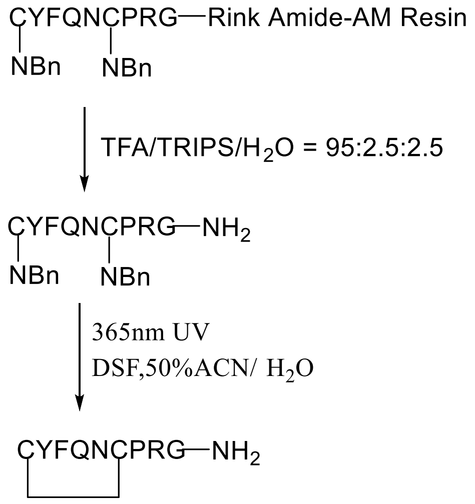

Figure S4 Synthesis of the peptide drug vasopressin

2.1 Preparation of Linear Vasopressin Precursor

The synthesis was carried out using Fmoc-SPPS on a Rink Amide-AM Resin (0.74 mmol/g, 0.25 mmol scale). Peptide synthesis was performed on peptide synthesizer in presence of 4 equiv. of amino acid, HBTU and 8 equiv. of N, N'-diisopropylethylamine (DIEA). 20% piperidine in DMF was used to remove the Fmoc-protecting group. After the reaction completed, we obtained the linear vasopressin precursor resin bearing two Cys (1&6) modified with 2-nitrobenzyl (NBzl). The linear vasopressin precursor resin was washed three times with DMF (10 ml/time). 10 ml of methanol was added to shrink the resin for 30 min, then methanol was suctioned away, and vacuum drying was performed to obtain 570 mg of resin.

570 mg of the linear vasopressin precursor resin was added to a 25 ml round bottom flask, 10 ml of a preformulated solution TFA/TRIPS/H_2O = 95:2.5:2.5 (*v*/*v*/*v*) was added and reacted at room temperature for 1.5 h. The resin was suction filtered and the filtrate was collected. The resin was washed with a small amount of TFA and the filtrates were combined. Then the filtrate was evaporated to 2 ml and the crude peptide was precipitated with precooled Et_2O (20 ml). The solid was washed three times with precooled Et_2O (20 ml /time), dissolved in ACN-H_2O (1:1) (25 ml), and lyophilized. The HPLC analysis was carried out on a C18 analytical column (4.6 × 100 mm) using a gradient of 0-50% B (0.1%TFA-acetonitrile) with 1 ml/min, the monitor wavelength was 214 nm. For preparative HPLC, C18 column (30 × 250 mm) in gradient of 0-50% B (0.1%TFA-acetonitrile) with 20 ml/min, the monitor wavelength was 214 nm, was used to provide the pure linear vasopressin precursor peptide.

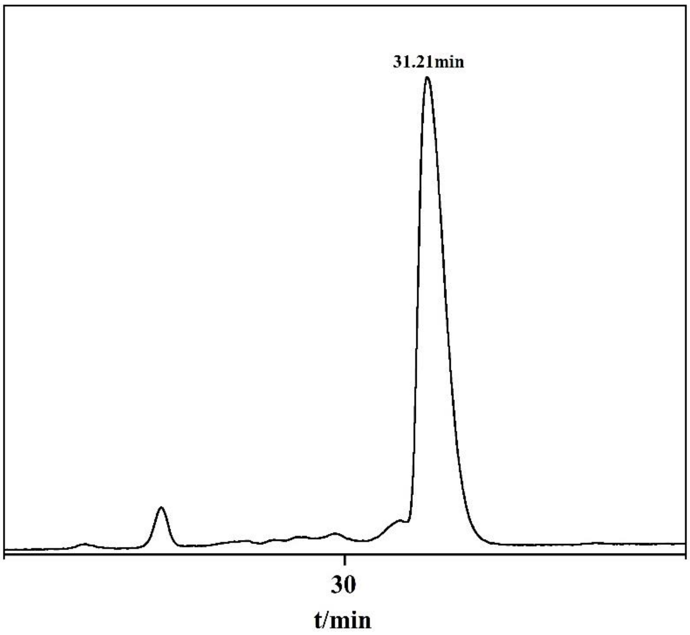

Figure S5 The HPLC spectrum of linear vasopressin precursor peptide

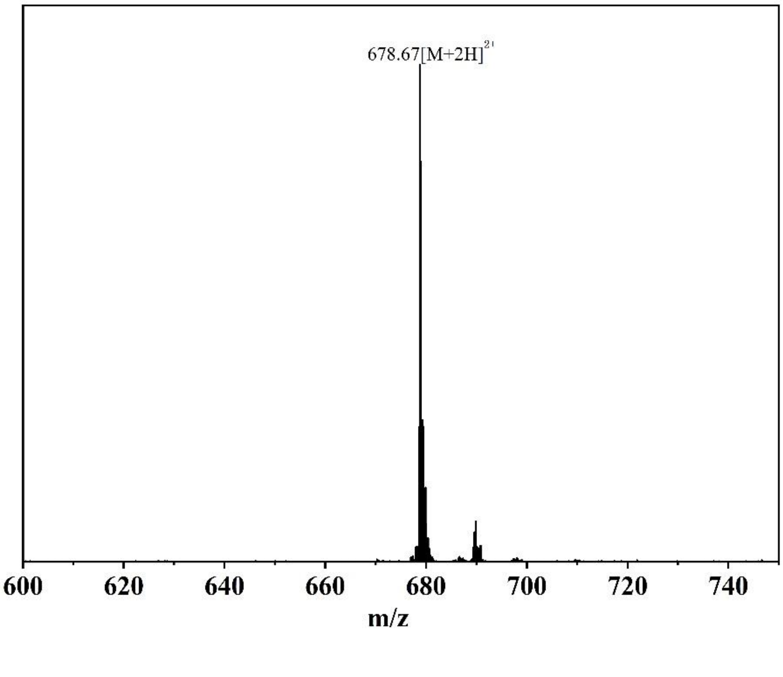

Figure S6 The MS spectrum of linear vasopressin precursor peptide

2.2 Preparation of Vasopressin

2-nitrobenzyl (o-NBn) photosensitive PG of Cys can induce a fast (within 8 min) and simultaneous decaging and selective disulfide formation when it's exposed to UV light in presence of DSF at pH 7. Thus, we tried to optimize the reaction conditions to formation the disulfide bond of vasopressin. In order to improve

ultraviolet light reaction efficiency, buffer, oxidizing agent and UV wavelength were investigated (Table S1). The result of experiments showed that the optimal reaction condition was DSF as the oxidation reagent, 50% ACN/H_2O as buffer and exposed to 365 nm UV light (Table S1, entry 7).

Table S1 Optimizing reaction conditions for formation of the third disulfide bond from UV light reaction.

Entry	Buffer	Oxidizing Agent	UV wavelength	Yield
1	6M Gn·HCl	DSF	full wavelength	32%
2	6M Gn·HCl	DSF	365nm	41%
3	6M Gn·HCl	H_2O_2	full wavelength	15%
4	6M Gn·HCl	H_2O_2	365nm	15%
5	50 mM NH_4HCO_3	air	365nm	0
6	0.1%TFA/H_2O	DSF	365nm	0
7	50%ACN/ H_2O	DSF	365nm	75%
8	50%ACN/ H_2O	H_2O_2	365nm	47%
9	50%ACN/ H_2O	DSF	full wavelength	29%

To a 5 ml round bottom flask was sequentially added linear vasopressin precursor peptide (1mg, 1 μmol), DSF (225 μg, 10 μmol) and 1.5 ml 50% ACN/H_2O. The mixture was directly exposured to UV irradiation at 365 nm for 25 min (the reaction liquid level was 5 cm away from the light source). Subsequently the mixture was continued to react for 2 h at room temperature without UV light source. Purification was performed by HPLC using semi-preparative C18 column (10 × 250 mm) in gradient of 0-50% B (0.1%TFA-acetonitrile) with 2 ml/min, the monitor wavelength was 214 nm. Target peak fractions were collected, concentrated and lyophilized to give 75% yield.

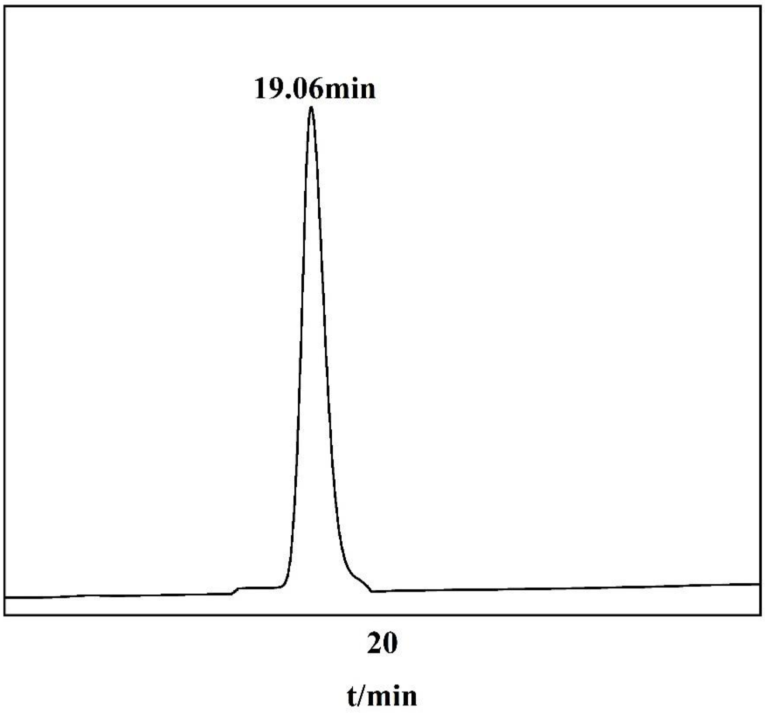

Figure S7 The HPLC spectrum of vasopressin peptide

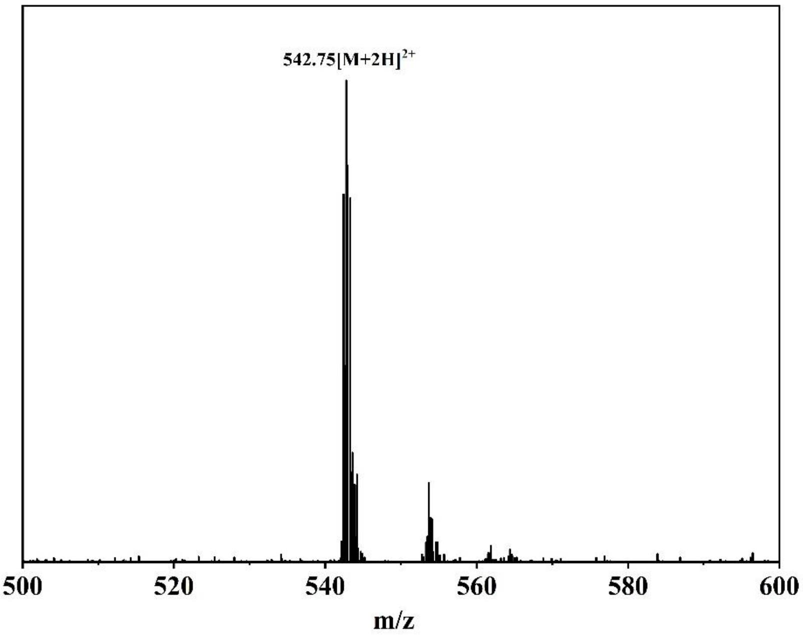

Figure S8 The MS spectrum of vasopressin peptide
